# Supplementary material for: Causal Relationship between Aging and Anorexia Nervosa: A White-Matter-Microstructure-Mediated Mendelian Randomization Analysis
Source: Biomedicines. 2024 Aug 16;12(8):1874. doi: 10.3390/biomedicines12081874 (PMC11351342; doi:10.3390/biomedicines12081874)
Supplement: Supplementary file 1 [file biomedicines-12-01874-s001.zip › Supplementary Figure/Figure S4/Singe SNP plot of MR analysis for TL to ubm-b-1673.pdf]

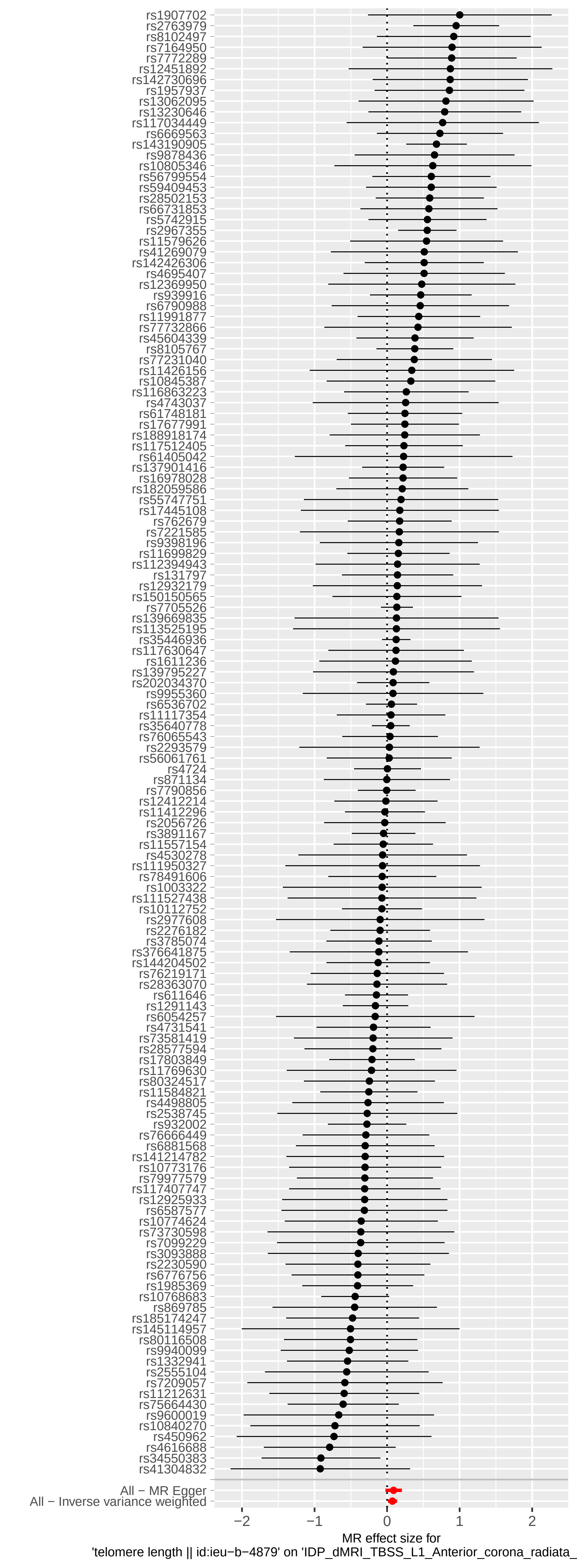

rs1907702
rs2763979
rs8102497
rs7164950
rs7772289
rs12451892
rs142730696
rs1957937
rs13062095
rs13230646
rs117034449
rs6669563
rs143190905
rs9878436
rs10805346
rs56799554
rs59409453
rs28502153
rs66731853
rs5742915
rs2967355
rs11579626
rs41269079
rs142426306
rs4695407
rs12369950
rs939916
rs6790988
rs11991877
rs77732866
rs45604339
rs8105767
rs77231040
rs11426156
rs10845387
rs116863223
rs4743037
rs61748181
rs17677991
rs188918174
rs117512405
rs61405042
rs137901416
rs16978028
rs182059586
rs55747751
rs17445108
rs762679
rs7221585
rs9398196
rs11699829
rs112394943
rs131797
rs12932179
rs150150565
rs7705526
rs139669835
rs113525195
rs35446936
rs117630647
rs1611236
rs139795227
rs202034370
rs9955360
rs6536702
rs11117354
rs35640778
rs76065543
rs2293579
rs56061761
rs4724
rs871134
rs7790856
rs12412214
rs11412296
rs2056726
rs3891167
rs11557154
rs4530278
rs111950327
rs78491606
rs1003322
rs111527438
rs10112752
rs2977608
rs2276182
rs3785074
rs376641875
rs144204502
rs76219171
rs28363070
rs611646
rs1291143
rs6054257
rs4731541
rs73581419
rs28577594
rs17803849
rs11769630
rs80324517
rs11584821
rs4498805
rs2538745
rs932002
rs76666449
rs6881568
rs141214782
rs10773176
rs79977579
rs117407747
rs12925933
rs6587577
rs10774624
rs73730598
rs7099229
rs3093888
rs2230590
rs6776756
rs1985369
rs10768683
rs869785
rs185174247
rs145114957
rs80116508
rs9940099
rs1332941
rs2555104
rs7209057
rs11212631
rs75664430
rs9600019
rs10840270
rs450962
rs4616688
rs34550383
rs41304832
All − MR Egger
All − Inverse variance weighted
−2
−1
0
1
2
MR effect size for
'telomere length || id:ieu−b−4879' on 'IDP_dMRI_TBSS_L1_Anterior_corona_radiata_
